# Cancer and SARS-CoV-2 Infection: Diagnostic and Therapeutic Challenges

**DOI:** 10.3390/cancers12061581

**Published:** 2020-06-15

**Authors:** Alessandro Allegra, Giovanni Pioggia, Alessandro Tonacci, Caterina Musolino, Sebastiano Gangemi

**Affiliations:** 1 Division of Haematology, Department of Human Pathology in Adulthood and Childhood “Gaetano Barresi”, University of Messina, 98125 Messina, Italy; cmusolino@unime.it; 2 COVID Centre AOU Policlinic G. Martino Messina, 98125 Messina, Italy; 3 Institute for Biomedical Research and Innovation (IRIB), National Research Council of Italy (CNR), 98164 Messina, Italy; giovanni.pioggia@cnr.it; 4 Clinical Physiology Institute, National Research Council of Italy (IFC-CNR), 56124 Pisa, Italy; atonacci@ifc.cnr.it; 5 School and Operative Unit of Allergy and Clinical Immunology, Department of Clinical and Experimental Medicine, University of Messina, 98125 Messina, Italy; gangemis@unime.it

**Keywords:** cancer, SARS-CoV-2, COVID-19, epidemiology, treatment, prognosis, risk factor, immunosuppression, cytokines

## Abstract

In late December 2019, a new infectious viral disease appeared. A new betacoronavirus, severe acute respiratory syndrome coronavirus 2 (SARS-Cov-2), has been recognized as the pathogen responsible for this infection. Patients affected by tumors are more vulnerable to infection owing to poor health status, concomitant chronic diseases, and immunosuppressive conditions provoked by both the cancer and antitumor therapies. In this review, we have analyzed some lesser known aspects of the relationship between neoplasms and SARS-CoV-2 infection, starting from the different expression of the ACE2 receptor of the virus in the various neoplastic pathologies, and the roles that different cytokine patterns could have in vulnerability to infection and the appearance of complications. This review also reports the rationale for a possible use of drugs commonly employed in neoplastic therapy, such as bevacizumab, ibrutinib, selinexor, thalidomide, carfilzomib, and PD-1 inhibitors, for the treatment of SARS-CoV-2 infection. Finally, we have highlighted some diagnostic challenges in the recognition of SARS-CoV-2 infection in cancer-infected patients. The combination of these two health problems—tumors and a pandemic virus—could become a catastrophe if not correctly handled. Careful and judicious management of cancer patients with SARS-Cov-2 could support a better outcome for these patients during the current pandemic.

## 1. Introduction

The World Health Organization (WHO) has lately stated that the present pandemic of coronavirus disease 2019 (COVID-19) is a public health crisis of international interest [1]. As of May 27—5,500,000 COVID-19 infected patients had been registered globally, and more than 300,000 people had died from the virus, now known as severe acute respiratory syndrome coronavirus 2 (SARS-CoV-2).

With more than 18 million new cases per year worldwide, cancer affects a considerable proportion of the global population, and subjects affected by tumors are more vulnerable to infections owing to poor health status, concomitant chronic diseases, and immunosuppressive conditions provoked by both the tumors and antitumor therapies [2,3]. In addition, apart from altered host defenses, other factors, such as leukopenia, disturbance to the barriers to infection, and changes in microbial flora, could be important [4,5,6].

As a consequence, patients with tumors who are infected by the SARS-CoV-2 coronavirus may suffer worse outcomes than other subjects. In fact, in a previous outbreak of a related virus which gave rise to the Middle East Respiratory Syndrome epidemic in 2015, a mortality rate of 84% was observed in tumor-affected subjects, which was twice as high as the rate in non-oncology subjects [7].

Similarly, throughout the influenza A virus subtype H1N1 (H1N1) epidemic in 2009, mortality for cancer subjects hospitalized with H1N1 was up to 18.5% higher [8]. However, although the H1N1 epidemic was protracted, it did not have the same impact as the SARS-CoV2 epidemic appears to be having, nor was it as fatal.

It has been predicted that at least 6270 supplementary deaths could occur in England over the next 12 months in subjects with new tumor diagnoses, as an effect of the SARS-CoV-2 outbreak [9].

### Incidence and Severity of SARS-CoV2 Infection in Cancer Patients

According to analyses of subjects receiving treatment for SARS-CoV-2 in Chinese hospitals, those with tumors appear to be at greater risk of infection and tend to have less positive prognoses.

Liang et al. reported on 2007 cases from 575 hospitals. A total of 18 (1%) of 1590 SARS-CoV-2 subjects had a history of tumors, which is higher than the occurrence of tumors in the general Chinese population (0.29%). Lung tumors were the most common form (28%). Of the subjects with tumors and SARS-CoV-2, 25% had been subjected to chemotherapy or surgery within the past month, while 75% of subjects were cancer survivors in follow up after resection. Compared to subjects without tumors, subjects with tumors were older (mean age 63·1 years vs. 48·7 years), had more polypnea (47% vs. 23%), were more likely to have a history of smoking (22% of patients vs. 7%), and had more severe baseline computed tomography signs (94% vs. 71%). There were no relevant variations in comorbidities, sex, or baseline gravity of X-rays. Most notably, subjects with tumors had a greater risk of critical incidents (patients admitted to an intensive care unit (ICU), invasive ventilation, or death) with respect to subjects without tumors (39% vs. 8%). These data were validated by logistic regression after adjusting for other risk factors. Cancer history was the greater risk factor for critical events. Among subjects with tumors, older age was a risk factor for critical events. Subjects with lung tumors did not have a greater possibility of critical events with respect to subjects with different tumor forms. Moreover, utilizing a Cox regression model to analyze the time-dependent risk of developing critical events, it was demonstrated that subjects with tumors worsened more quickly than those without tumors (median time to critical events: 13 vs. 43 days [10] (Table 1)).

In an analogue study, Yu et al. calculated the infection percentage of SARS-CoV-2 in subjects with tumors from a single institution at 0.79% (12 of 1524 patients). Seven of the 12 (58.3%) subjects had non-small cell lung carcinomas (NSCLC). Five (41.7%) were being given chemotherapy with or without immunotherapy or radiotherapy. Three patients (25.0%) had a severe respiratory syndrome; one subject needed to be admitted to the intensive care unit [13].

These data were confirmed by Chen’s paper, which proposed that among those infected with SARS-CoV-2, about 1% of subjects had tumors (3): this was 5-fold greater than the overall occurrence of cancer in China (201.7/100,000 persons) [14,21].

Other authors have re-analyzed the greatest case series described by the Chinese center for disease control and prevention (44,672 cases), to evaluate the clinical risk factors correlated with death. Tumors (RR = 2.926, 95%CI = 1.34–6.41) were the main risk factors for mortality of subjects with SARS-CoV-2 [15].

A similar multi-center analysis was performed by Dai et al., employing subject information gathered from 14 hospitals in Hubei Province, China, in which they reported the clinical features and outcomes (development of severe respiratory syndrome, invasive mechanical ventilation, ICU admission, and death) of infected patients for 105 hospitalized subjects with tumors and 536 subjects without tumors. The data demonstrated SARS-CoV-2 subjects with cancer had greater risks for all severe outcomes. Subjects with hematological neoplasia, lung cancer, or metastatic cancer had the greatest incidence of critical events. Non-metastatic cancer subjects presented with similar rates of critical events to subjects without tumors. Patients who received surgery had higher risks of having critical events, while subjects submitted only to radiotherapy did not display relevant differences in critical events with respect to patients without cancer [22].

An updated WHO report reported a mortality of 7.6% among patients with cancer [1], while according other studies, cancer subjects are at a higher risk of critical events in (48–54% of cases vs. 16% in the general population) and death (5.6–29% vs. 3.4% in the general population) [11].

The results on the frequency of SARS-CoV-2 infection in tumor subjects seem diverse, and effects greater than those reported so far may be found when this is evaluated in other geographic areas. Miyashita et al. examined the electronic medical records (EMR) of Mount Sinai Health System (MSHS) in New York City from 1 March to 6 April, 2020, using Epic SlicerDicer software. They evaluated relevant clinical and demographic information (age, sex, comorbidities, critical events, and death rate) from 5688 patients with SARS-CoV-2, and found 334 subjects (6%) with tumors in this group (57 breast, 56 prostate, 23 lung, 18 urothelial, and 16 colon cancer). Without correcting for age groups, subjects with tumors were intubated considerably more frequently, but the death rate was not significantly different. After stratifying subjects by age, they demonstrated a significantly augmented risk of intubation in subjects with tumors if aged 66–80. Unexpectedly, subjects with tumors who were younger than 50 years had a higher mortality percentage. Moreover, the mortality of SARS-CoV-2 in tumor subjects was lower than that in subjects without tumors in age groups older than 50 years, although the difference was not statistically significant. The uncertain connection between SARS-CoV-2 and intubation or death is a drawback in this analysis. Moreover, the heterogeneity of tumor forms and diverse stages of cancers may provoke some uncertainty in the interpretation of the data [20].

In contrast to data from China and the USA, a report from Italy suggested that 20% of subjects with SARS-CoV-2 had been treated for a tumor in the previous 5 years. However, in this study, the detection of comorbidities was limited to subjects who died of SARS-CoV-2; therefore, differentiating between tumors as an independent risk factor for developing SARS-CoV-2 or as a risk factor for a bad prognosis is not possible [17].

In a different study performed on 355 Italian subjects, 20.3% had an active tumors, in addition to several other comorbidities. Although a higher mean age (79.5 ± 8.1 years) was registered within this group, separate analyses have demonstrated a death percentage estimated at 20% in subjects aged 80 and older [18].

A specific analysis was performed on particular subsets of patients, such as those affected by onco-hematological pathologies. One might suppose subjects with immune system tumors, such as lymphomas and lymphoid leukemias, might be at augmented risk for contracting SARS-CoV2 with respect to patients with myeloid cancers, such as Acute Myeloid Leukemia and Myelodysplastic Syndromes (AML and MDS), but an evaluation found no such connection. He et al. performed a cohort study at two centers in Wuhan, China, of 128 hospitalized patients with hematological neoplasia, 10% of whom contracted SARS-CoV-2, and of 226 health care providers, 16 of whom contracted SARS-CoV-2 and 11 of whom were hospitalized. The case rate for SARS-CoV-2 in hospitalized subjects with hematological diseases was 10%, compared to 7% in health care providers. Nevertheless, the 13 patients with hematological diseases had more serious SARS-CoV-2, and more from this group died from the illness. The case fatality percentages were 62% and 0%, respectively [16].

The augmented case fatality rate of patients with hematological disorders and SARS-CoV-2 appears to be correlated essentially to bacterial infections. This is consistent with a greater possibility of reduced granulocyte levels because of their disease or treatment.

Regarding the gravity of the infection in cancer patients and the high mortality rate in cancer patients, it should be borne in mind that patients with SARS-CoV-2 may struggle to overcome critical phases such as intubation; full intensive care support and life sustaining therapies cannot overcome the poor prognosis for certain high-risk populations affected by SARS-CoV-2 [23,24,25].

A separate discussion must then be had in the field of pediatric oncology. Hrusak et al. performed a flash survey on SARS-CoV-2 occurrence and gravity among children on antitumor treatments. They collected data from 25 countries, and about 10,000 patients considered at risk were followed. Over 200 of these children were tested, nine of whom were positive for SARS-CoV-2. Eight of the nine cases had an asymptomatic to mild infection [12].

The low percentage of infection in this population is rather unexpected, as it is reasonable to presume that pediatric subjects with tumors would be at least as vulnerable to infection as their healthy peers. SARS-CoV-2 does infect children in general, although the minor gravity of the infection increases the likelihood of underreporting in children.

The mild infection experienced by three children in this report was in contrast to a formerly reported case. An 8 year old child undergoing myelosuppressive chemotherapy for T-cell acute lymphoblastic leukemia in ALL in a Wuhan hospital presented with respiratory failure, necessitating mechanical ventilation. During the disease, CRP and Interleukin (IL)-6) were only slightly increased, but ferritin concentrations were elevated (6417–15,758 ug/L). This is evocative of characteristics of hemophagocytic lymphohistiocytosis, which has been reported to co-occur with infections [26].

A description of the Italian experience at a major childhood cancer center in Lombardy has recently been presented [19]. This report described five positive cases in subjects with childhood tumors, all of whom had a benign course and survived. Three subjects were treated at home, and two in hospital.

As for a possible explanation for the different course that tends to occur in children, the quantitative and qualitative changes in cytokine delivery can cause a diverse immune responses, and may explain the diverse outcomes in specific patients. In fact, it has now been documented that pregnant women and children generally have a mild disease after SARS-CoV-2 infection, if not a fully asymptomatic one [27]. These types of subjects have an immune response skewed toward a T-helper Type 2 cells (TH2) profile (controlled by the TH2 cells), with a particular production of cytokines like IL-4 and IL-10, while production of the pro-inflammatory cytokines in SARS-CoV-2 infection is characteristic of TH1 cells.

This specific immunological setup could somewhat defend pediatric tumor subjects from the most critical clinical manifestations of the infection.

Despite the presence in the literature of sometimes discordant data, this discordance can probably be linked to the low number of samples collected, the heterogeneity of neoplastic pathologies, and ethnic differences. It is likely that cancer patients have a different susceptibility to SARS-CoV-2 infection, a different course, and a different prognosis.

## 2. Pathophysiology of SARS-CoV 2 Infection in Cancer Patients

**Figure 1:**
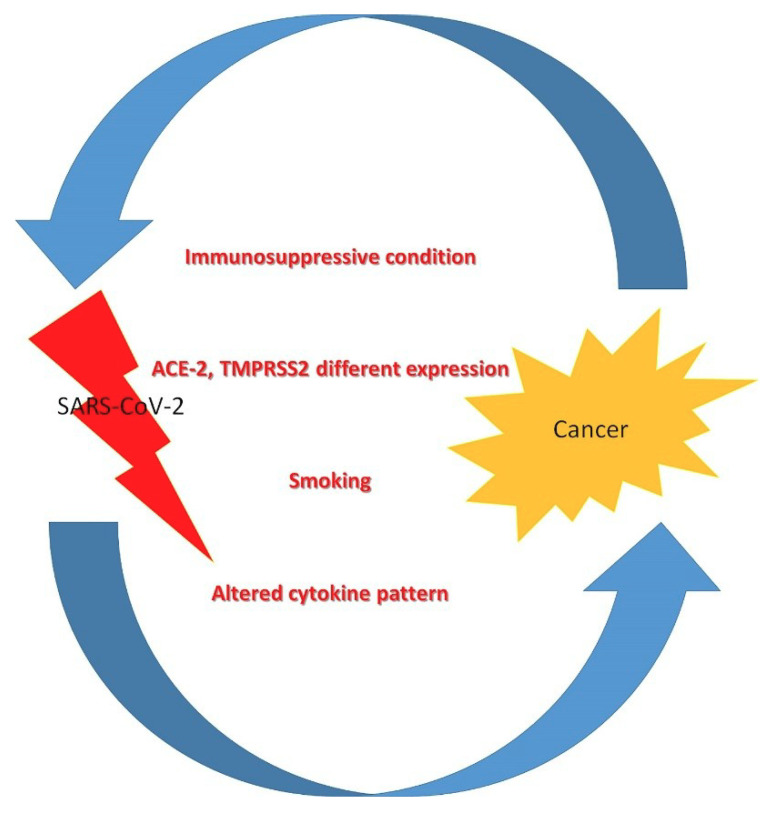
Pathophysiology of severe acute respiratory syndrome coronavirus 2 (SARS-CoV-2) infection in cancer patients.

The present data remain inadequate to explicate an irrefutable correlation between tumors and SARS-CoV2 infection or poorer outcomes. However, some considerations and hypotheses can be formulated on the subject (Figure 1).

SARS-CoV2’s cellular entry receptor, angiotensin-converting enzyme 2 (ACE2) [28], may be overexpressed in some tumors, including in pancreatic, cervical, and renal cancers [29]. However, other experiments have suggested the expression of ACE2 is considerably reduced in prostate, liver, and breast tumors, with respect to normal contiguous tissues.

Specific studies have been conducted in patients with renal papillary cell carcinoma and endometrial cancer.

ACE2 is a component of the renin-angiotensin system, however the relationship between ACE2 and prognosis in UCEC (uterine corpus endometrial carcinoma) and KIRP (kidney renal papillary cell carcinoma) is not well-defined. ACE2 is augmented in KIRP and UCEC, and increased ACE2 is associated with a favorable outcome. The expression of ACE2 appears to be positively correlated with the amount of immune infiltration of macrophages in KIRP, CD4+T cells, B cells, neutrophils, and dendritic cell immune infiltration in UCEC. Increased ACE2 expression is correlated with a favorable prognosis in UCEC and KIRP. What is more, it has been demonstrated that the expression of ACE2 is reduced in vivo and in vitro after SARS-Co-V2 infection. In conclusion, cancer may make sufferers more vulnerable to SARS-CoV-2 infection in the cases of KIRP and UCEC, leading to a poorer prognosis [30].

Similar studies have been conducted in lung cancer, as these patients appear to be more susceptible to infection than normal subjects [31].

The gene expression level of ACE2 may indicate susceptibility to SARS-CoV-2 infection. Transmembrane serine protease 2 (TMPRSS2) has a supportive action.

In fact, SARS-CoV-2 infected hACE2 transgenic mice showed serious pulmonary alterations, comprising interstitial hemorrhage, protein exudation, lymphocytic infiltration, and alveolar epithelial cell growth [32]. SARS-CoV-2 infected TMPRSS2-KO mice displayed reduced inflammatory cytokine reactions to intranasal stimulation. TMPRSS2 reduction modified the primary sites of infection and the virus spread within the airways, leading to a less critical immunopathology [33].

In another study, the correlation between gene expression of ACE2 and TMPRSS2 and outcomes in lung adenocarcinoma (LUAD) and lung squamous cell carcinoma (LUSC) was explored [34]. Lung tumor subjects in each subtype and stage were found to be predisposed to SARS-CoV-2 infection, excluding the primitive subtype of LUSC. LUAD subjects are more vulnerable to SARS-CoV-2 infection than LUSC subjects. TMPRSS2 may be a tumor suppressor gene, as it was severely reduced in LUAD and LUSC.

Indeed, the relationship between ACE2, cancer, and smoking may have importance, and a diverse possible justification for different vulnerabilities and outcomes in lung cancer subjects might be the greater smoking history in these patients [35]. Experimental findings have demonstrated that tobacco use considerably augments the gene expression of ACE2, which could lead to increased susceptibility to SARS-CoV2 in smokers [36]. Moreover, cigarette smoking is the main cause of chronic obstructive pulmonary disease, which has been recognized as an independent risk factor in critical SARS-CoV2 patients [37,38].

In addition to the above, although not all tumor subjects should be considered equally immunocompromised, tumors are always connected with an increased expression of immunosuppressive cytokines, decreased pro-inflammatory danger signals, and an augmented functional immunosuppressive leukocyte population, which may cause a dampened immune system and augment the probability of infectious complications [39].

Some specific cytokines may favor SARS-CoV2 infection, and at the same time have a role in neoplastic diseases. For instance, IL-17 could be central to this process. In the lung, the IL-17 cytokine is generated by TH17 cells in reaction to viruses. IL-17 stimulates signaling, which in turn induces the production of chemokines. These substances enroll immune system cells to the inflammation site. The permanency of the virus causes a hyperactivity of the immune system which can provoke a cytokine storm [40], and IL-17 stimulates, in synergy with IL-6, viral permanency by stopping apoptosis [41]. Other research has found that it is possible to clarify the biological mechanisms correlated with organ injury due to virus action via the IL-17 pathway [41]. In addition to provoking a cytokine storm and blocking the programmed cell death of infected cells, it also appears to have the capability to augment the proliferation of some viruses by augmenting their virulence [42]. It has further been reported, in an experimental model, that viral persistence, by producing a persistent augmentation of IL-17, provokes ARDS (acute distress respiratory syndrome) [43], just as it occurs in SARS-CoV-2 infection. The augmented concentration of IL-17 could also be correlated with the hypercoagulation condition sometimes seen in SARS-CoV-2 subjects [44].

Moreover, an elevated amount of TH17 lymphocytes released into the alveolar space has been described. At the same time, the role of IL-17 is well known in lung cancer, as IL-17 stimulates VEGF production in cancer cell lines [45]. This action is supported by the STAT3-Gα–Interacting Vesicle-Associated Protein (GIV) pathway, and is stopped when cells are exposed to small interfering RNA (siRNA) [46]. It has been reported that patients with augmented concentrations of IL-17 were less likely to survive and had increased angiogenesis with respect to healthy controls [47]. Similarly, exposure of diverse NSCLC cell lines to IL-17 increased neo-angiogenesis and augmented in vivo cancer proliferation in SCID mice via a C-X-C chemokine receptor (CXCR-2)-dependent mechanism. In fact, IL-17 increased numerous pro-angiogenic CXC chemokines, including CXCL1, CXCL5, CXCL6, and CXCL8. Blocking IL-17 with monoclonal antibodies abrogated this up-regulation, and could be theoretically advantageous in subjects with SARS-CoV-2 infection.

Anti-IL-17 use is well established and approved in psoriatic arthritis, and has demonstrated a therapeutic action not only in several tumor forms [48], but also in the therapy of lung infections due to the H1N1 virus [47], ARDS [49], and pulmonary fibrosis [50]. If a study was able to determine that IL17 target therapy can both control tumor diseases and lead to resolution of SARS-CoV-2 infection, it could be applied as a treatment for SARS-CoV-2 patients with lung cancer [51].

## 3. Diagnostic Challenges in the Recognition of SARS-CoV-2 Infection and Neoplastic Disease in Cancer-Infected Patients

The presence of a neoplastic disease can complicate some diagnostic elements of SARS-CoV-2 infection, such as respiratory distress syndrome. Both SARS-CoV-2 and tumor non-SARS-CoV-2 conditions, such as superior vena cava obstruction, lung metastasis, or upper airway tumors, can provoke breathlessness and distress. The presenting characteristics of SARS-CoV-2, such as fever, fatigue, and dyspnea, are frequently analogous to those of subjects with tumors, particularly those undergoing therapy. Therefore, the identification of SARS-CoV-2 symptoms in such subjects can be difficult (Table 2).

Not only can the clinical appearance of SARS-CoV-2 in tumor subjects be unique, but identifying this disease is problematic due to several factors. For instance, tumor subjects might have atypical radiographic features. Qu et al. A study described the case of one subject affected by lung adenocarcinoma who had a laboratory established SARS-CoV-2 infection with irregular, diffuse, small ground-glass opacities and partial consolidation on chest CT-scan. This is not congruent with the typical peripheral subpleural ground-glass infiltrates seen in this infection [52]. Lung tumor subjects might have radiographic features alike to those of a SAR-CoV-2 infection, and this might be misleading. A study described the cases of five of 139 tumor subjects who had ground-glass opacities on baseline chest CT-scans. Three of these subjects submitted to RT-PCR, had a negative test, and were judged negative for SARS-CoV-2 [53].

Moreover, many cancer markers, such as carbohydrate antigens (CA) and carcinoembryonic antigens (CEA), increase during several inflammatory situations in the lungs. Wei et al. postulated that SARS-CoV-2-provokes acute lung damage, which may be accompanied with augments of some tumor markers [54]. In this retrospective analysis, the concentrations of several serum biomarkers were performed in SARS-CoV-2 subjects (mild: 131; severe: 98; critical: 23). They reported that there were relevant augments in concentrations of carcinoembryonic antigen (CEA), carbohydrate antigens (CA) 125 and 153, human epididymis protein 4 (HE4), and cytokeratin-19 fragment (CYFRA21-1) in SARS-CoV-2 mild cases with respect to normal controls; their concentrations exhibited constant and relevant augments in severe and critical cases. CA199 and squamous cell carcinoma antigen (SCC) are augmented considerably only in critical cases of SARS-CoV-2, with respect to mild and severe cases and normal controls. There were positive correlations between concentrations of C-reactive protein and concentrations of CEA, SCC, HE4, CYFRA21-1, CA153, and CA125 [54]. In this analysis, the authors excluded any subjects with tumor diagnoses; therefore, the increase of these cancer markers was not correlated with pre-existing situations of tumorigenesis. Moreover, numerous previous studies have established that cancer biomarkers are also elevated in various inflammatory conditions in the lungs [55,56,57,58,59,60] (Table 2).

Finally, other biological and clinical characteristics can disguise SARS-CoV-2’s presentation in tumor subjects. For instance, hematologic malignancies can cause laboratory results to be misleading.

The presence of one of the two pathologies, or both in the same subject, can therefore make the diagnostic process more difficult.

## 4. Antineoplastic and Antiviral Therapy in Infected Patients with Neoplasia

**Figure 2:**
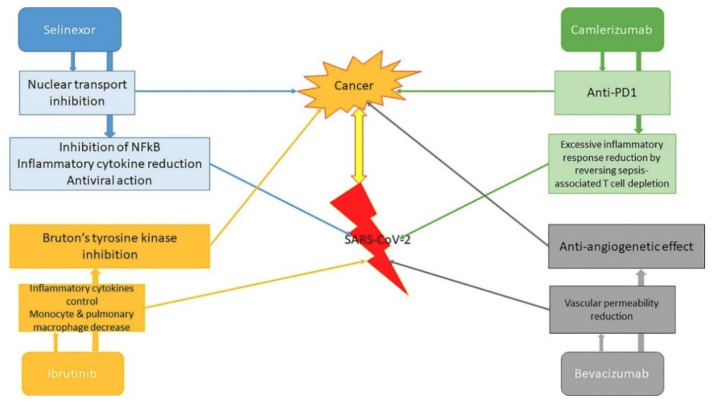
Antineoplastic and antiviral actions of drugs commonly used in various neoplastic diseases. PD-1: programmed death-1.

The aim of this review was not to analyze in detail the therapy of individual neoplastic pathologies in patients with SARS-CoV-2, nor to suggest how the presence of infection should change the approach to neoplastic disease. As we will briefly mention later, specific national and international oncology societies have issued dozens of comprehensive guidelines capable of guiding the diagnostic, therapeutic, and follow-up interventions of health professionals. In the next paragraphs we will evaluate instead those drugs used in the oncology field that can interfere with the clinical evolution of SARS-CoV-2 infection (Figure 2).

For instance, Chinese trials are currently evaluating the action of the anti-vascular endothelial growth factor (VEGF) bevacizumab on SARS-CoV-2 patients (NCT04275414). VEGF is the most potent vascular permeability inducer, and higher levels of VEGF have been found in SARS-CoV-2 patients compared with healthy controls. Other studies will try to evaluate the effects of drugs, such as the myeloma drug thalidomide (NCT04273581), the programmed death-1 (PD-1) inhibitor camrelizumab (NCT04268537), and other anti-TNF tNFkumor drugs in the therapy of SARS-CoV-2 infection. The possible value of other targeted substances, such as carfilzomib, afatinib, and ixazomib, will also be assessed [61].

Regarding molecules that target the immune check points PD-1 and its ligand (PD-L1), these drugs have changed the therapy and outcome of numerous forms of tumors. These monoclonal antibodies are able to restore antitumor immunity, and have received approval for the treatment of several forms of tumors, including lung cancer, breast cancer, melanoma, and urological tumors [62,63,64,65].

The immune-related adverse events (irAEs) of anti-PD-1 or anti-PD-L1 substances are generally inflammation against organ-specific targets. Patients can present with irAEs such as pneumonitis, hepatitis, myocarditis, nephritis, and encephalitis. There is no augmented occurrence of hepatitis in patients with chronic hepatitis or immune reconstitution in subjects with HIV infection [66,67]. However, there have been cases of reactivation of latent tuberculosis via an augment in the immune response [68]. Reports of fatal encephalitis or myocarditis found in Epstein–Barr virus positive lymphocytes in the affected histological region have been released, suggesting some role of the infection in this irAE [69]. However, differently to chemotherapy, which is immunosuppressive, immune checkpoint inhibitors may be a safer possibility, as one case series of tumor subjects with SARS-CoV-2 infection did not report any cases among those submitted to immunotherapy [10]. Thus, patients may be less inclined towards grave infections, but are at risk of a cytokine release syndrome that would aggravate a SARS-CoV-2 infection [70,71,72]. PD-1 blockade is anticipated to reduce the development of sepsis secondary to severe pneumonia and excessive inflammatory response syndrome in COVID-19 patients, by reversing sepsis-associated T cell depletion.

However, the management must be cautiously calculated on a case-by-case scenario, given the possible augmentation in the risk of side-effects or death from the SARS-CoV-2 infection [73]. Checkpoint inhibitors (ICIs) appear to be more supportable than other chemotherapeutic drugs [74,75]. If the studies on camrelizumab in patients with SARS-CoV-2 infection give comforting results [61], the use of this drug could be an important element for the treatment of the disease in many neoplastic patients.

A different substance that seems to have surprising implications for subjects with SARS-CoV-2 infection is ibrutinib. Ibrutinib is a powerful covalent inhibitor of Bruton tyrosine kinase (BTK). Ibrutinib is also a strong reversible inhibitor of hematopoietic cell kinase (HCK).

The possibility for ibrutinib to reduce lung damage, pulmonary inflammatory cytokines, and death has in the past been established in a lethal flu experimental animal model, in which animals were challenged with an intranasal inoculum of H1N1 influenza virus. Control animals presented with respiratory failure, with histological and Computed Tomography (CT) features coherent with relevant lung damage, in contrast to the animals treated with ibrutinib. Non-treated animals died, while those treated with ibrutinib recuperated and regained their weight after an initial loss, and all survived [76].

Some experimental investigations have allowed elucidation of the mechanisms of action of ibrutinib. It was demonstrated that BTK, and its activator HCK, are implicated in Toll-like receptor (TLR) mediated signaling [77,78,79]. Both BTK and HCK are stimulated by Myeloid differentiation factor 88 (MyD88), a TLR-adaptor protein that acts on all Toll receptors excluding TLR3 in response to viruses, including coronaviruses [80]. ATII cells have TLRs, as do the alveolar macrophages that regulate inflammatory responses. As central elements of TLR/MYD88 signaling, BTK and HCK can control inflammatory cytokine generation via Extracellular signal-regulated kinases (ERK)1/2 [81].

In a transgenic animal model, stimulation of HCK caused serious pulmonary inflammation and an increase of the innate immune response, especially in older animals [82]. Augmented concentrations of TNF-a were found in the bronchoalveolar lavage fluids of these animals. The pulmonary alterations evidenced in these mice looked very similar to those reported in the lungs of subjects with SARS-CoV-2 infection, which presented with fibrin exudation with alveolar infiltration of monocytes and macrophages [83].

Treon et al. tried to elucidate the effect of ibrutinib in SARS-CoV-2 subjects [84]. They evaluated 300 Waldenstrom’s macroglobulinemia (WM) patients treated with a BTK-inhibitor. They investigated six subjects receiving ibrutinib (five patients received 420 mg/day; one patient received 140 mg/day) who were diagnosed with SARS-CoV-2 infection. Their median time with SARS-CoV-2 correlated symptoms prior to diagnostic analysis was five days. All six subjects had fever and a cough as prodromal signs. The five patients on ibrutinib at 420 mg/day had no dyspnea, and did not need hospitalization. Their course was characterized by constant improvement, and disappearance of SARS-CoV-2 correlated symptoms. The subject on treatment with ibrutinib 140 mg/day presented with increasing dyspnea and hypoxia, provoking hospitalization. Chest CT demonstrated bilateral ground glass opacities. This induced a hold on ibrutinib administration, with a severe worsening of hypoxia [84].

Although the study included only a small series of patients, it surely offers interesting possibilities. Subjects on ibrutinib may benefit from maintenance of their treatment, despite the presence of SARS-CoV-2 infection, although it will be essential to confirm these data in other subjects on BTK-inhibitors, including CLL patients.

Low-dose selinexor will be evaluated in a randomized clinical trial for hospitalized subjects with severe SARS-CoV-2 infection (NCT04349098). This oral drug has been approved for the treatment of subjects with relapsed/refractory multiple myeloma [85]. It is a selective inhibitor of nuclear export (SINE) compound that acts on cellular protein Exportin (XPO1). The protein supports the transportation of numerous essential proteins from the nucleus to the cytoplasm, increasing the actions of pro-inflammatory transcription factors. SINE elements have also shown the capability to reduce the proliferation of several viruses, such as RNA viruses, influenza, and respiratory syncytial virus, in vitro and in vivo. Moreover, these elements have been able to provoke anti-inflammatory and anti-viral activities in several animal models, including showing effects in respiratory infections. Finally, SINE compounds have been demonstrated to be able to inhibit relevant host protein interactions with SARS-CoV-2 [86].

## 5. The Management of Cancer Patients with SARS-CoV2 Infection

The present SARS-CoV-2 pandemic is modifying how we face tumors in several ways. In the context of SARS-CoV-2, greater importance is being placed upon the therapy of cancer patients who may be at augmented risk for having serious and potentially fatal events [87].

Cytotoxic therapies employed for hematological malignancies can provoke a reduction of lymphocyte subsets, possibly making subjects more vulnerable to infection [88,89].

Analogous reflections must be formulated for the employment of radiotherapy. This type of treatment may worsen myelosuppression and augment the risk of SARS-CoC-2 infection. Nevertheless, there are several possibilities for radiotherapy timing, and suitable single fraction or short courses of radiation are recommended [90].

Although the effects of viral pandemics on tumor subjects are far from certain, the choice to continue with anti-tumor treatment, including surgery, during the conditions of the SARS-CoV-2 outbreak should be cautiously evaluated given the possibility of immunosuppression, critical respiratory complications, or death. An analysis of 34 asymptomatic subjects who undertook diverse elective surgeries during the course of the SARS-CoV-2 incubation period helped to clarify these risks [91]. All 34 subjects developed SARS-CoV-2 pneumonia just after surgery (median time to SARS-CoV-2 onset, 3.5 days), with 44% requiring ICU admission. Half of these subjects died in the ICU.

However, delaying specific tumor surgeries may be correlated with an augmented risk of progression.

Several solid cancers, such as pancreatic or lung cancer and some hematologic diseases, such as acute leukemia, require urgent therapy. However, other early-stage tumors, such as prostate, cervical, breast, and nonmelanoma skin tumors, may be able to be treated with a certain delay.

Consequently, the risk:benefit ratio of a systemic antitumor therapy has to be evaluated. For each cancer subject, numerous elements, such as performance status, age, comorbidities, and the number of hospital visits required for the therapy, can modify this risk [92].

As a result of the pandemics, elective medical care has been de-prioritized in many places, and a logical framework to evaluate patient prioritization is required. Oncology societies and national authorities have been quick to release guidelines on tumor care during the outbreak [93].

For example, the NHS in England has defined a prioritization of systemic anticancer treatments, as displayed in Table 3.

Chemotherapy in a local hospital is suggested to decrease population movement. High-dosage chemotherapy or protocols with relevant myelotoxicity should be avoided, while Granulocyte Colony-Stimulating Factor (G-CSF) should be administered. Oral chemotherapy drugs suitable for home-based therapy are preferred, particularly for palliative therapy.

The creation of a mathematical framework might permit a quantitative analysis to balance conflicting risks and support treatment decision making. Incorporation of SARS-CoV-2-correlated risk models into the analysis of randomized trials could guide clinical decisions during this outbreak [95].

## 6. Conclusions

SARS-CoV-2 is seriously interfering with tumor therapy, and nullifying attempts to treat cancer patients adequately [96].

The combination of these two health problems, tumors and the outbreak, could become a catastrophe if not correctly handled. Mishandling could affect thousands of lives and lead to wasted time, money, and energy. The ultimate objective of all treatment options is to decrease the damage as much as possible [97].

Moreover, the negative effects of the outbreak on tumor patients are not restrained to direct effects only. This pandemic has reduced the activities of several research centers and oncological clinical units, has stopped patient enrolment into clinical trials, and has halted novel clinical studies. Most trials need supplementary visits and tests, further augmenting the possibility for diffusion of infection. Finally, the amount of new drugs able to be released, both in terms of production and delivery, may be reduced [98].

Careful and judicious management of cancer patients with SARS-Cov2 could guarantee a better outcome for these patients, and a less dramatic impact due to the current pandemic.

## Figures and Tables

**Table 1:** Incidence and severity of severe acute respiratory syndrome coronavirus 2 (SARS-CoV-2) in cancer patients worldwide.

Study	Subjects	Findings
**All/various countries/continents**		
Worldometer (2020) [11]	All confirmed worldwide cases (>5 million)	Critical events in 48–54% of cancer patients vs. 16% in general population; death in 5.6–29% vs. 3.4%
Hrusak et al. (2020) Various countries [12]	10,000, of which 2000 children analyzed	4.5% with tumor, of which 88.9% asymptomatic or mildly symptomatic
**Asia**		
Liang et al. (2020) China [10]	2007	Tumor history in SARS-CoV vs. general population: 1% vs. 0.29% Subjects with tumor worsened more quickly (13 vs. 43 days until critical events) and were more critical (39% vs. 8%)
Yu et al. (2020) China [13]	1524	0.79% with tumor in SARS-CoV 25% of those with tumor had severe respiratory syndrome, 8.3% were admitted to intensive care unit (ICU)
Chen et al. (2020) China [14]	99	Tumor history in SARS-CoV vs. general population: 1% vs. 0.2%
Deng et al. (2020) China [15]	44,672	Relative Risk=2.926 for tumors as risk factors for fatality of patients with COVID-19
WHO (2020) China [1]	75,465	7.6% of mortality for cancer patients, fifth highest after cardiovascular disease (13.2%), diabetes (9.2%), hypertension (8.4%), chronic respiratory disease (8.0%)
He et al. (2020) China [16]	354	10% with tumor in SARS-CoV-2 in general public (7% in healthcare providers) More severe SARS-CoV in patients with haematological diseases
**Europe**		
Palmieri et al. (2020) Italy [17]	37,860	20% with tumor in SARS-CoV-2
Onder et al. (2020) Italy [18]	355	20.3% with tumor in SARS-CoV-2 Mortality = 20% of subjects with tumor older than 80
Balduzzi et al. (2020) Italy [19]	5 children	100% survival (60% were paucisymptomatic)
**Americas**		
Miyashita et al. (2020) USA [20]	5688	6% with tumor in SARS-CoV Higher complications, higher mortality in younger patients with cancer

**Table 2:** Common features, markers and clinical characteristics between SARS-CoV-2 and tumor non-SARS-CoV-2.

Common Features of SARS-CoV-2 and Tumor non-SARS-CoV-2	Common Markers of SARS-CoV-2 and Tumor non-SARS-CoV-2	Other Clinical Characteristics Common in SARS-CoV-2 and Tumor non-SARS-CoV-2
Superior vena cava obstruction Lung metastasis Upper airway tumors Breathlessness Distress Fever Fatigue Dyspnea	Carbohydrate antigens Carcinoembryonic antigen Human epididymis protein 4 Cytokeratin-19 fragment CYFRA21-1 Squamous cell carcinoma antigen (only critical cases)	Hematologic malignancies

**Table 3:** Prioritizing systemic anticancer treatments (extracted and adapted from National Health System England’s clinical guide for the management of non-coronavirus patients requiring acute treatment: cancer [94]).

Priority Level	Treatment
1	Curative treatment with high chance of success (>50%) Adjuvant/neoadjuvant treatment which adds at least 50% chance of cure to surgery/radiotherapy alone/treatment given at relapse
2	Curative treatment with an intermediate chance of success (20–50%) Adjuvant/neoadjuvant treatment which adds 20–50% chance of cure to surgery/radiotherapy alone/treatment given at relapse
3	Curative treatment with a low chance of success (10–20%) Adjuvant/neoadjuvant treatment which adds 10–20% chance of cure to surgery/radiotherapy alone/treatment given at relapse Non-curative treatment with a high chance of more than 1 year extension (>50%)
4	Curative treatment with a very low chance of success (0–10%) Adjuvant/neoadjuvant treatment which adds less than 10% chance of cure to surgery/radiotherapy alone/treatment given at relapse Non-curative treatment with an intermediate chance of more than 1 year extension to life (15% to 50%)
5	Non-curative treatment with a high chance of palliation or temporary tumor control (>50%) and less than 1 year expected extension to life
6	Non-curative treatment with an intermediate chance of palliation or temporary tumor control (15–50%) and less than 1 year expected extension to life
